# A Three-Stage Voltage Adjustment Method for Correcting the Inconsistent Action of the Inchworm Piezoelectric Structure

**DOI:** 10.3390/ma19214756

**Published:** 2026-11-09

**Authors:** Xinfu Deng, Zicheng Qin, Tian Qiu, Yuqing Jian, Yixiao Yang

**Affiliations:** 1 College of Integrated Circuits and Micro-Nano Electronics, Fudan University, Shanghai 200433, China; 2 Yunnan Key Laboratory of Coffee, Yunnan Agricultural University, Pu’er 665099, China; 3 Shanghai Engineering & Research Center of Ultra-Precision Motion Control and Measurement, Fudan University, Shanghai 200433, China

**Keywords:** inchworm piezoelectric structure, inconsistent action, material dispersion, hysteresis asymmetry, contact timing differences, three-stage voltage adjustment method

## Abstract

The macro stage of the tea-coffee nanoscale detection device adopts an inchworm piezoelectric structure. However, this structure has the phenomenon of inconsistent action, affecting the motion accuracy. To study this phenomenon, the causes of actuation errors were first analyzed, including material dispersion, hysteresis asymmetry, and contact timing differences. Based on this, a three-level voltage adjustment method was then proposed. This method sequentially performs three steps of correction: actuation capability balance, shear hysteresis compensation, and lateral consistency alignment. It systematically improves the consistency of the inchworm piezoelectric structure’s action. Experimental results show that the three-level voltage adjustment method increases the single-step displacement consistency of the actuator by approximately 28.72%, and also significantly improves the stability of the overall movement.

## 1. Introduction

The tea-coffee nanoscale detection device is a tropical agricultural science and technology equipment integrating piezoelectric material actuation and optical detection technologies. Its motion platform adopts a coarse-fine configuration. The macroscopic motion is realized by the periodic actuation of inchworm piezoelectric structures for coarse positioning over a long travel range, whereas the microscopic motion is achieved through direct-drive actuation using flexible hinge structures for nanometer-scale fine positioning [1,2]. These motion components possess the typical advantages of piezoelectric actuators, including high precision, fast response, and non-magnetic operation [3,4]. In addition, the use of inchworm piezoelectric structures provides long-stroke capability, making the system suitable for low-speed and high-resolution movements and satisfying the analytical and detection requirements of tea and coffee testings in agricultural and food-related applications [5,6].

The error of the direct-drive actuation in the tea-coffee nanoscale detection device mainly originates from the inherent hysteresis nonlinearity of the piezoelectric material, and extensive research has been conducted on this issue of similar device. Lin et al. proposed a Bouc–Wen hysteresis model identification method based on joint optimization, in which the model parameters were calibrated through an objective function [7]. Wang et al. combined a Prandtl–Ishlinskii (PI) model incorporating preload parameters with second-order finite-element analysis, improving the nonlinear prediction accuracy of flexible piezoelectric structures by more than 80% [8]. Baziyad et al. combined a modified Preisach hysteresis model for piezoelectric actuators with $H_{\infty }$ robust control, which denotes the infinity norm associated with the Hardy space of stable and proper transfer functions [9]. With continued iterative development, research on direct-drive actuation has progressed rapidly beyond single-model identification and compensation, and hysteresis-induced positioning errors have been effectively addressed [10,11,12].

On the other hand, the nanoscale detection platform is achieved through the periodic coordinated actuation of longitudinal stacks and shear stacks in inchworm piezoelectric structure [13]. The resulting errors are primarily associated with the structure characteristics, and this issue has been found and considered in several studies. Tian et al. tested different frequencies on a U-shaped stepping structure with two piezoelectric feet and found that frequency and velocity did not exhibit a directly linear relationship [14]. Cai et al. developed an inchworm piezoelectric motor with separated longitudinal and shear actuation, collected displacement characteristics at different velocities using variable-frequency speed regulation, and observed differences in displacement capability under different loads [15]. To avoid errors caused by frequency variation in inchworm piezoelectric structures, Yang et al. employed an improved frequency-control adjustment to correct motor errors, reducing the open-loop positioning error rate from 7.38% to 0.393%. This work provided an initial feasible control strategy for inchworm-like piezoelectric structures [16].

Comparing with the eliminated influence of hysteresis on direct-drive motion compensating by models, the periodic motion error of the macro-stage is generally solved by enhancing global tracking accuracy. Existing studies cannot adequately account for single-step motion accuracy [17]. The single-step actuation of each piezoelectric stack in the inchworm piezoelectric structure has a significant influence on macro-stage’s action consistency. Unanalyzed disturbance factors associated with mechanical engineering include:

1. Differences in the inherent actuation capability of the PZT materials, which result in a “long-leg/short-leg” effect among multiple piezoelectric stacks;
2. Geometrical errors and fitting clearances introduced during manufacturing and assembly, which alter the expected contact timing between the piezoelectric stacks and the mover.

In addition, asymmetry in the hysteresis characteristics of individual piezoelectric elements can also affect contact timing [18,19]. These motion inconsistencies are details that cannot be adequately addressed by current studies. Therefore, a three-stage voltage adjustment method is proposed to address the above issues. The method ensures the accuracy of contact timing and actuation displacement from three aspects: actuation capability correction, shear nonlinearity compensation, and opposite-side consistency alignment. Consequently, motion errors of the macro-stage can be reduced and the periodic action in the inchworm piezoelectric structures can be made more stable.

The remainder of this paper is organized as follows. Section 2 quantitatively analyzes the causes of macro-stage motion errors associated with material dispersion, hysteresis asymmetry, and contact timing difference in inchworm piezoelectric structures using mathematical models. Section 3 proposes a three-stage voltage adjustment method based on the above analysis. After acquiring parameters from structural assembly, the actuation control system sequentially performs actuation capability correction, shear nonlinearity compensation, and opposite-side consistency alignment through control-voltage adjustment. Section 4 presents experiment on inchworm piezoelectric structures, in which the motion results before and after adjustment are recorded, compared, and analyzed with respect to the desired displacement. Section 5 concludes this study.

## 2. Analysis of Inconsistency

**Figure 1:**
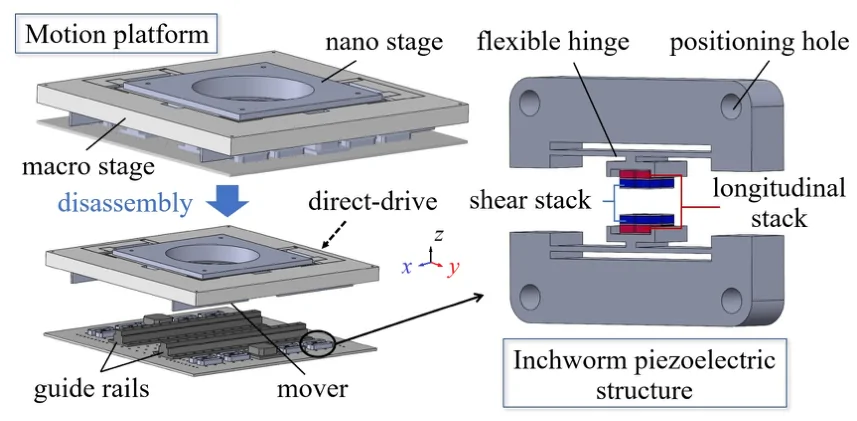
Structure of the motion platform of the tea and coffee nanoscale detection device.

Figure 1 illustrates the structure of the motion platform of the tea and coffee nanoscale detection device. This section analyzes the formation of macro-stage motion errors using mathematical models associated with material dispersion, hysteresis asymmetry, and contact timing difference.

### 2.1. Analysis of Material Dispersion

**Figure 2:**
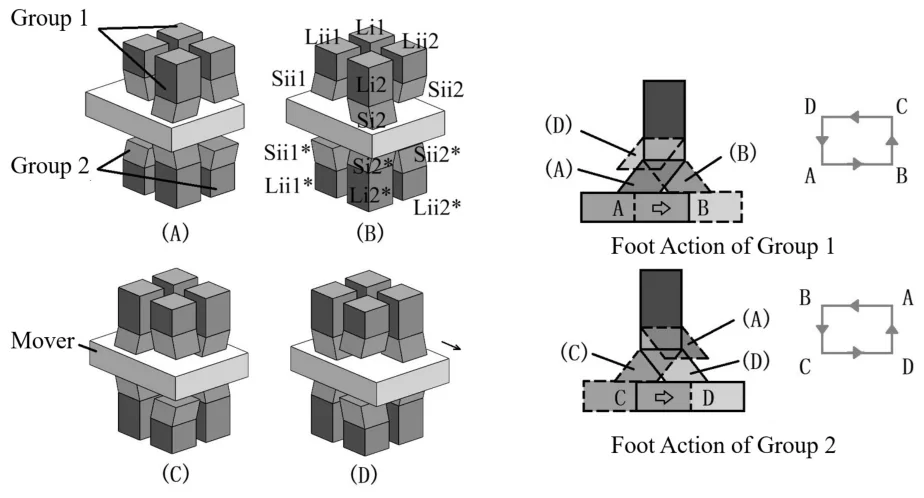
Motion diagram of stepping strategy in the inchworm piezoelectric structures. *: the symmetricpositions in the lower half are marked with an asteris. (A): sequence A; (B): sequence B; (C): sequence C; (D): sequence D.

The inchworm piezoelectric structure consists of multiple piezoelectric stacks that are driven in groups to achieve an inchworm-like walking motion. Eight inchworm piezoelectric structures are placed horizontally and symmetrically in device. The deformation direction of the shear stack is *x*, the deformation direction of the longitudinal stack is *y*, and the vertical direction of the device is *z*. As a commonly used stepping strategy illustrated in Figure 2, the driving units of the inchworm piezoelectric structure are divided into two groups, with four piezoelectric feet in each group. The contacting part of the macro-stage can be regarded as the mover. Each piezoelectric foot consists of a longitudinal stack and a shear stack with equal cross-section area. The feet perform symmetric cyclic motions relative to the mover according to the sequence (A)–(B)–(C)–(D), thereby maintaining a balanced force on the mover.

The piezoelectric stacks in the inchworm piezoelectric structures can be assigned 16 identification numbers according to their actuation direction, group, and orientation. The longitudinal stacks are denoted by *L*, while the shear stacks are denoted by *S*. The stacks belonging to the first group are denoted by *i*, and those belonging to the second group are denoted by ii. The two different piezoelectric feet within each group are denoted by 1 and 2. The upper half of Figure 2 is labeled directly, whereas the symmetric positions in the lower half are marked with an asterisk.

In practical manufacturing, the material and fabrication parameters of the 16 piezoelectric stacks differ from one another. Consequently, their key performance parameters exhibit statistical material dispersion such as piezoelectric coefficient and elastic modulus, resulting in inconsistent action capabilities. As an example, under the same applied voltage *U*, the theoretical elongation $\delta_{i}$ of the *i* th piezoelectric stack is given by [20]

(1) $$\delta_{i}=d_{33,i}\cdot U.$$

where $d_{33,i}$ represents the piezoelectric constant. Since the piezoelectric constant differs among the stacks, the longitudinal and shear elongations of each piezoelectric foot in the inchworm piezoelectric structures are also inconsistent, ultimately producing the “long-leg/short-leg” motion characteristic. Its negative effects on the motion are mainly reflected in the following aspects:

1. Uneven preload distribution: During the clamping stage, a piezoelectric foot with a larger longitudinal elongation bears a greater mechanical load, whereas a shorter foot may exhibit insufficient contact. This is illustrated by the *L*-labeled piezoelectric stacks within the same group in Figure 2.
2. Uneven propulsion distance: During the propulsion stage, the shear elongation determines the effective propulsion distance of the mover. Differences in shear elongation directly result in displacement differences, sliding relative to the mover. This is illustrated by the *S*-labeled piezoelectric stacks within the same group in Figure 2.

The “long-leg/short-leg” effect has also been demonstrated in other experiments [15,21]. Therefore, when piezoelectric stacks within the same group are driven by the same voltage, differences in elongation inevitably occur, and the corresponding voltage values should be adjusted accordingly.

### 2.2. Analysis of Hysteresis Asymmetry

In addition to differences in elongation, the hysteresis characteristics of piezoelectric stacks also differ. Under hysteresis, the elongation of a piezoelectric stack depends not only on the instantaneous driving voltage but also on the historical voltage trajectory [22]. Even when the controller outputs the same voltage sequence, the actual displacement of each piezoelectric foot may differ because of differences in its initial state and previous motion history, resulting in inconsistent action.

The instantaneous actuation velocity $v_{i}$ of the *i*th piezoelectric stack is the derivative of its displacement $x_{i}$ with respect to time *t* [11]:

(2) $$v_{i}=\lim_{\Delta t\to 0}\frac{\Delta x_{i}}{\Delta t}=\frac{dx_{i}}{dt}.$$

Therefore, compared with differences in actuation capability caused by the piezoelectric coefficient, hysteresis mainly affects the actuation velocity of the piezoelectric stacks, as manifested by the following:

1. Contact-time offset: Under the same peak-to-peak voltage, the asymmetry between the ascending and descending branches of the hysteresis loop causes the longitudinal stacks to reach the contact position at different times. When the inchworm piezoelectric structure changes its direction of motion, the time at which the piezoelectric feet contact the mover is shifted.
2. Nonlinear action velocity: Hysteresis causes a nonlinear voltage–displacement response of the shear stacks. The resulting variation in mover velocity causes the *S*-labeled piezoelectric stacks within the same group to slide relative to the mover, thereby affecting the periodic action after motion direction switched.

**Figure 3:**
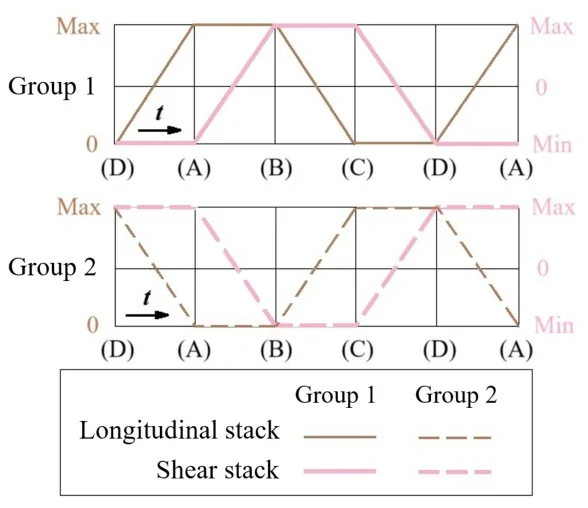
Driving voltage corresponding to the action state. (A): sequence A; (B): sequence B; (C): sequence C; (D): sequence D.

Figure 3 shows the driving voltage waveforms corresponding to the stepping sequence in Figure 2. The waveforms are based on the conventional full-step actuation strategy, in which the longitudinal stacks clamp and release the mover while the shear stacks provide the shear displacement. The horizontal axis represents the corresponding action state, while the vertical axis represents the voltage range [23]. The dashed and solid lines represent the voltage variation trends within one cycle for the two groups. The longitudinal and shear stacks are distinguished by different colors. The two voltage-holding intervals are retained to allow the current clamping or propulsion action to be completed before the next action begins. This sequencing helps avoid unintended overlap between successive actions and maintains the intended contact relationship between the piezoelectric legs and the mover.

The proportion of the actuation cycle assigned to each voltage-holding interval determines the duration available for the corresponding action. Changing this proportion changes the slope and duration of the voltage transitions and can therefore be used to regulate the effective forward-motion velocity. The waveforms are thus selected according to the mechanical sequence of the inchworm piezoelectric structure and the required speed regulation, rather than being arbitrary signal shapes.

The hysteresis characteristics of longitudinal and shear stacks are also been demonstrated in other experiments [19,24]. Under full-step strategy, the longitudinal stacks have sufficient time to contact the mover, hence their influence on the motion can be excluded. To avoid reverse-direction motion errors caused by hysteresis asymmetry from the shear piezoelectric stacks, the macro-stage controller needs to compensate for shear stack’s nonlinear characteristics.

### 2.3. Analysis of Contact Timing Difference

In addition to the material-related issues discussed above, assembly errors in mechanical engineering can also cause inaccurate motion of inchworm piezoelectric structures, including positional deviations, fitting clearances, and installation-surface parallelism errors. These errors are not only static offsets, but also converting into random delays or advances in contact timing. Although the four piezoelectric feet on one side can be aligned relatively easily, simultaneous contact during symmetric motion cannot necessarily be guaranteed on the opposite side, thereby disturbing the expected periodic coordinated action.

Let $t_{0}$ denote the nominal contact time and $t_{c}$ the actual contact time. To describe the possible timing deviations introduced by assembly tolerances, the contact time is expressed as

(3) $$t_{c}=t_{0}+\Delta t_{\alpha },\;\Delta t_{\alpha }∼N\left(0,\sigma_{c}^{2}\right).$$

where $\Delta t_{\alpha }$ is the contact timing deviation and $\sigma_{c}$ is the standard deviation associated with the assumed timing-error distribution. Under this modeling assumption, a positive $\Delta t_{\alpha }$ indicates delayed contact, whereas a negative value indicates earlier contact. The distribution is introduced to represent timing uncertainty in the analytical model; it should not be interpreted as an experimentally identified probability distribution unless supporting measurements are available.

The contact timing difference directly changes the relative velocity and insertion depth (assuming that the stacks are flexible) when the piezoelectric foot contacts the mover, causing the dynamic preload to deviate from its designed value:

(4) $$F_{\mathrm{pre}}=F_{0}\left[1+\beta \;\mathrm{sgn}\left(\Delta t_{\alpha }\right){\left|\Delta t_{\alpha }\right|}^{1/2}\right].$$

In this equation, $F_{\mathrm{pre}}$ denotes the dynamic preload during contact, $F_{0}$ is the nominal preload, and β is a model coefficient characterizing the assumed dependence of preload perturbation on contact timing deviation. The sign function specifies the direction of the perturbation, while the square-root term represents the assumed nonlinear dependence on the magnitude of the timing deviation. This expression is used as a phenomenological relation to illustrate how imperfect contact synchronization can alter the preload experienced by the piezoelectric stacks. When a contact timing difference exists, the longitudinal stack of the first contact piezoelectric foot experiences a greater preload than that on the opposite side, thereby it indirectly affecting the output capability of their shear stacks [8].

In addition, contact timing differences can indirectly deteriorate the control condition of the system. There may be several contact states such as double-foot and multi-foot contact between different groups or single-foot contact caused by premature contact, resulting in different identified mass, stiffness, and damping parameters of control system. The effect of contact timing difference must be eliminated before all the piezoelectric stacks put into real application. Otherwise, even if the segmented switching control method was proposed in previous studies, they cannot be effectively implemented [25]. In summary, the inchworm piezoelectric structures in the macro-stage require both correction of the elongation of piezoelectric feet within the same group and compensation of hysteresis asymmetry, as well as correction of the contact timing difference between opposite-side piezoelectric feet, to ensure that all piezoelectric stacks perform as expected.

## 3. Three-Stage Voltage Adjustment Method

Based on the analysis in Section 2, a three-stage voltage adjustment method combining scientific rationale with engineering applicability is proposed. Through a logically progressive proportion–model compensation–deviation adjustment procedure, the method eliminates action inconsistency in inchworm piezoelectric structures from the stack level to the global level.

### 3.1. Actuation Capability Correction

The first stage eliminates the “long-leg/short-leg” effect caused by material dispersion among the four piezoelectric feet on the same side, ensuring that the elongations of the corresponding stacks remain consistent under the same driving voltage. A standard elongation δ is first defined. Since the elongation of a piezoelectric stack is positively correlated with the applied voltage, under the same voltage *U* it requires:

(5) $$\delta =\delta_{i}\cdot k_{i}=d_{33,i}\cdot U\cdot k_{i}.$$

where $k_{i}$ is the voltage scaling coefficient used for adjustment. This coefficient does not change the driving voltage sequence shown in Figure 3; instead, it directly modifies the actual voltage applied to the piezoelectric stack. It can be obtained through experimental measurements and calculations, and implemented through the driving circuit.

In operation of the first adjustment stage, the integrated piezoelectric feet on one side are as a whole placed into a test mold. The mold keeps the piezoelectric feet fixed on one side, while a sensor on the other side measures their longitudinal displacement. Two movers are then inserted according to the group orientation, and the displacement measured by the sensors represents the shear displacement of the two different piezoelectric feet within the same group. Subsequently, the four piezoelectric feet are driven using the stepping strategy voltages shown in Figure 3, and the measurements are completed in sequence.

Based on the measurement results, the voltage scaling coefficient of a piezoelectric stack with weak actuation capability is set to 1. The voltage scaling coefficients of other piezoelectric stacks of the same type (longitudinal or shear) are calculated relative to this reference value. The resulting coefficients are then used to proportionally increase or decrease the driving voltage within each group until the elongations become consistent. The longitudinal stacks should be tested and adjusted before the shear stacks to ensure that the shear stacks experience equal preload when acting on the two movers in the test mold.

### 3.2. Shear Nonlinear Compensation

After single-side piezoelectric stacks have been adjusted in elongation, the inchworm piezoelectric structures must further ensure the consistency of the shear actuation velocities within the same group. Therefore, a hysteresis model is employed to compensate for the nonlinear characteristics and resolve the velocity synchronization problem caused by differences in hysteresis characteristics.

The Preisach model is adopted to describe the hysteresis relationship between the voltage input U(t) and the output δ(t) of a piezoelectric stack [9]:

(6) $$\delta \left(t\right)={\;\int \int \;}_{\alpha \geq \beta }\mu \left(\alpha ,\beta \right)\gamma_{\alpha ,\beta }\left[U\left(t\right)\right]\;d\alpha \;d\beta .$$

where μ(α,β) is the Preisach weighting function, and $\gamma_{\alpha ,\beta }$ is the hysteresis operator, whose output is either 0 or 1 as the input changes. α and β are the upper and lower switching thresholds, respectively, satisfying α>β. As distribution μ(α,β) can form an asymmetric hysteresis loop, the Preisach model accurately describes the memory dependence of the output on the input history.

In real practice of the second adjustment stage, the test mold containing two movers are used obtain the shear hysteresis characteristics. The hysteresis response of each shear stack is modeled separately, and its weighting function is identified. This function uniquely corresponds to a specific hysteresis path. Since the longitudinal stacks have already been adjusted in the first stage, the preload applied to the two movers during shear displacement testing is identical. The nonlinear characteristics of each shear stack are compensated by changing the applied voltage through feedforward control. Within the same cycle duration, the velocity of the linearized motion becomes consistent, hence relative sliding of the macro-stage is consequently avoided.

### 3.3. Opposite-Side Consistency Alignment

The inchworm piezoelectric structures are horizontally installed in device and are not affected by the gravitational force of the macro-stage. Once the guide-rail position has been accurately established, the inchworm piezoelectric structures can be reinstalled into the moving component and done the third adjustment stage by testing the macro-stage. During testing at a low actuation frequency, the macro-stage can clearly detect the two successive contacts of symmetrically positioned piezoelectric feet serving as the mover. The relationship between the contact time difference and the elongation offset $\delta_{\alpha }$ required for advance actuation is given by

(7) $$\delta_{\alpha }=\Delta t_{\alpha }\frac{4\delta_{\max}}{T},$$

where $\delta_{\max}$ is the total elongation of one longitudinal stack during a single actuation and *T* is the actuation cycle period. Here it is assuming that an approximately uniform elongation rate over this interval. This approximation is used to estimate the required compensation from the measured contact timing difference. Its accuracy depends on the validity of the quarter-cycle assumption and the approximately uniform elongation-rate assumption. Since the single elongation process is completed within one-quarter of the cycle, the elongation offset can be directly estimated from the actuation velocity. Taking the earlier contact as the reference, the elongation offset is generally positive. To correct the assembly error, the voltage offset $U_{\alpha ,i}$ of the longitudinal stack is

(8) $$U_{\alpha ,i}=\frac{\delta_{\alpha }}{d_{33,i}}.$$

This equation shows that the actual voltage offset still depends on the piezoelectric coefficient formulation. Since the preceding stages have already performed proportional adjustment and linearized the output of the shear stacks under equal preload, only the longitudinal stacks in opposite position need to be shifted in the third adjustment stage. The adjusted driving voltage $U_{i}\left(t\right)$ is therefore

(9) $$U_{i}\left(t\right)=U_{\alpha ,i}+U_{i,0}\left(t\right),$$

where $U_{i,0}\left(t\right)$ is the original driving voltage. Within the same group, the voltage on the side that contacts first remains unchanged, whereas the voltage on the opposite side is adjusted.

**Figure 4:**
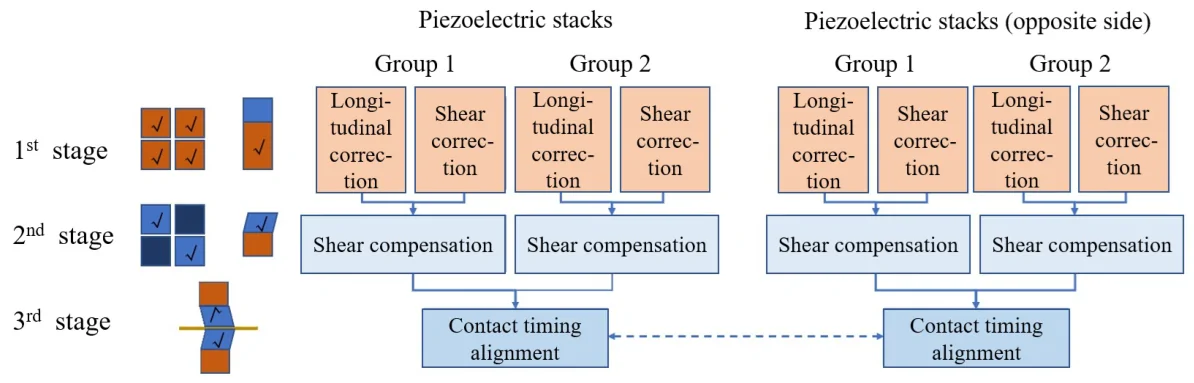
Process of the three-stage voltage adjustment method for inchworm piezoelectric structures.

The complete three-stage voltage adjustment procedure is summarized in Figure 4. The method tunes the actual driving voltage using proportional coefficients, a Preisach hysteresis model, and voltage offsets, thereby ensuring stable motion of the inchworm piezoelectric structure in every cycle.

## 4. Experiments and Results

### 4.1. Experimental Setup

**Figure 5:**
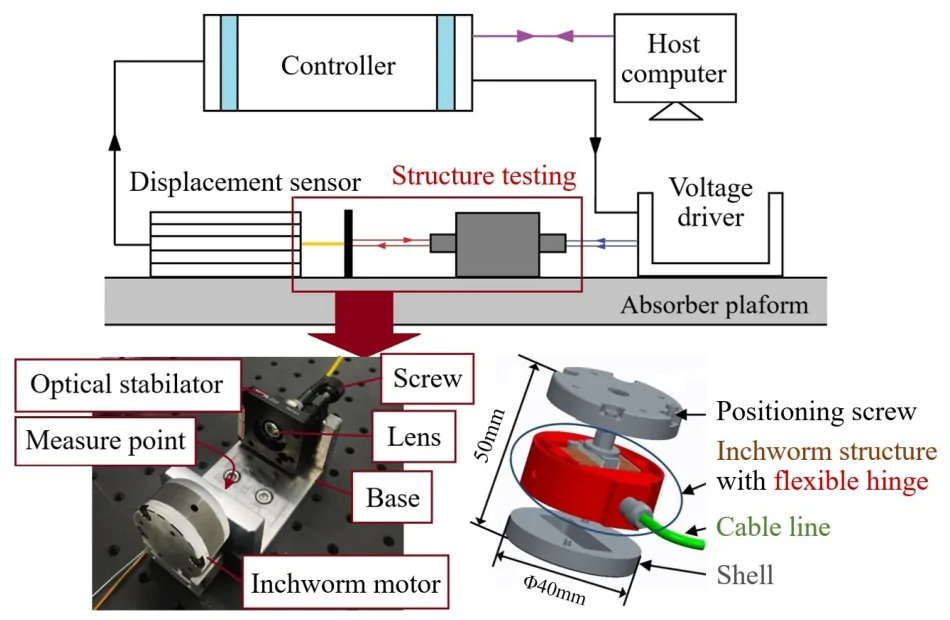
Diagram of experimental setup.

To verify the effectiveness of the proposed adjustment method, an experimental platform was constructed. The platform mainly consists of an inchworm piezoelectric structure test platform, a host computer, a voltage driver (amplification factor: 27.65), a displacement sensor (laser interferometer, Attocube IDS 3005, attocube systems AG, Haar, Germany), and a controller (Speedgoat real-time controller). The experimental object is an inchworm motor employing the same type of inchworm piezoelectric structure, as shown in Figure 5 [26].

The action principle of the inchworm motor is identical to that of the macro stage in the tea and coffee nanoscale detection platform. Also, the material (PZT), section area (5 mm × 5 mm), and length (7.5 mm) of the piezoelectric stacks of the inchowrm motor are exactly the same as those used in the device. The lightweight mover of the inchworm motor replaces the large mass macro stage, substantially reducing the size of the driving component. Moreover, the original inner grating ruler of the macro-stage is replaced by a higher-precision laser interferometer as displacement sensor. After optical alignment, the output displacement of the motor can be directly measured by the laser interferometer. The interferometer provides the displacement signal used to evaluate the motion before and after adjustment, with a sampling frequency of 1 MHz and the resolution of 0.1 nm. The host computer is used to configure the driving parameters and process the exported displacement data, where the data transfer to resolution of 100 nm and sampling frequency of 1 kHz to reduce the file size within observable range. The Speedgoat controller generates the voltage commands and outputs them through its analog-output interface. The commands are amplified by the voltage driver before being applied to the individual piezoelectric stacks.

**Figure 6:**
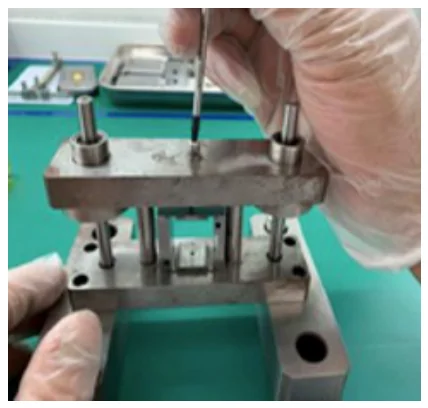
Adjustment mold.

This experiment adopts the similar framework as previous studies [17]. Compared with the four groups of parallel driving circuits used before, the present experiment employs 16 independent driving-voltage sets for eight longitudinal stacks and eight shear stacks, thereby providing sufficient degrees of freedom for adjusting motion consistency. First, the mover displacement of the unadjusted inchworm piezoelectric structure was measured at frequencies of 5 and 10 Hz. The amplitude parameter and action’s ratio parameter in one cycle of the original driving voltages are listed in Table 1. These are the reasonable values selected within the commonly used range (0.5–100 Hz, −150–150 V). The measured data were written into the controller through the host computer, amplified proportionally by the voltage driver, and then applied to the inchworm motor. The displacement data were exported from the laser interferometer software, processed, and plotted on host computer screen. Subsequently, the inchworm piezoelectric structure was adjusted using the mold according to the three-stage voltage adjustment operation procedure, as shown in Figure 6. The mold is an auxiliary fixture used during the offline adjustment procedure, rather than a structural component of the complete inchworm piezoelectric motor. The piezoelectric legs are positioned in the mold to constrain their installation orientation and facilitate the measurement and adjustment of their longitudinal and shear displacements. The mold therefore supports the practical implementation of the three-stage voltage adjustment procedure, but it is not involved in the operating configuration of the motor after calibration.

The voltage scaling coefficients and the parameters of the Preisach model were first calculated. The piezoelectric stacks after two adjustment stages were then reinstalled into the inchworm motor, after which the voltage offsets were calculated in the third stage. Finally, the displacement of the inchworm piezoelectric structure after the three-stage voltage adjustment method were tested at the same frequencies and compared with the unadjusted data. Since the calculations are straightforward and involve multiple parameters, the intermediate results are not presented in parameter table in this section; instead, they are analyzed by final plots.

### 4.2. Experimental Results

**Figure 7:**
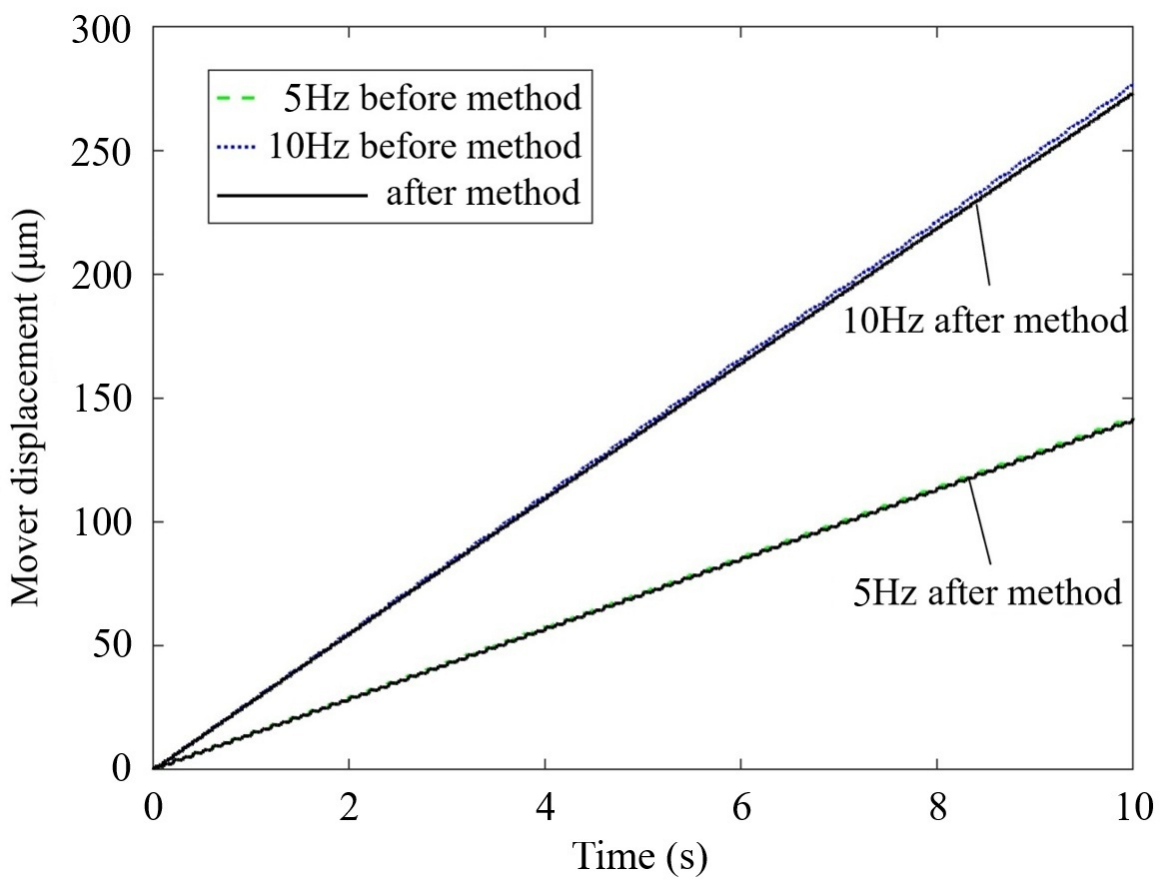
Comparison of displacement before and after the adjustment method.

The time–displacement characteristics of the inchworm piezoelectric structure measured at the two different frequencies are shown in Figure 7. The blue dash-dotted line represents the unadjusted displacement at 10 Hz, the green long-dashed line represents the unadjusted displacement at 5 Hz, and the black solid line represents the displacement after adjustment.

The experimental displacement is evaluated against the prescribed motion target for each operating condition. The desired displacement refers to the line from 0 to final displacement specified by the experimental trajectory (before adjustment), whereas the measured displacement represents the actual mover position recorded by the laser interferometer. To distinguish the prescribed target from the measured response, the corresponding desired displacement and the measured displacement change over the observation interval are summarized in Table 2. The comparison is intended to document the observed motion under the tested conditions, rather than to establish statistical repeatability across independent experiments.

Due to the periodic motion of the inchworm piezoelectric structure, the global displacement linearity after adjustment shows no significant difference from that before adjustment in 10 s. The total travel after adjustment is slightly lower than that before adjustment. This is attributed to the fact that the shortest-elongation piezoelectric foot, whose voltage scaling coefficient was set to 1 during adjustment, impacts on the average elongation of all feet, resulting in an accumulated difference over the entire travel. This difference did not have a significant effect on the mover.

**Figure 8:**
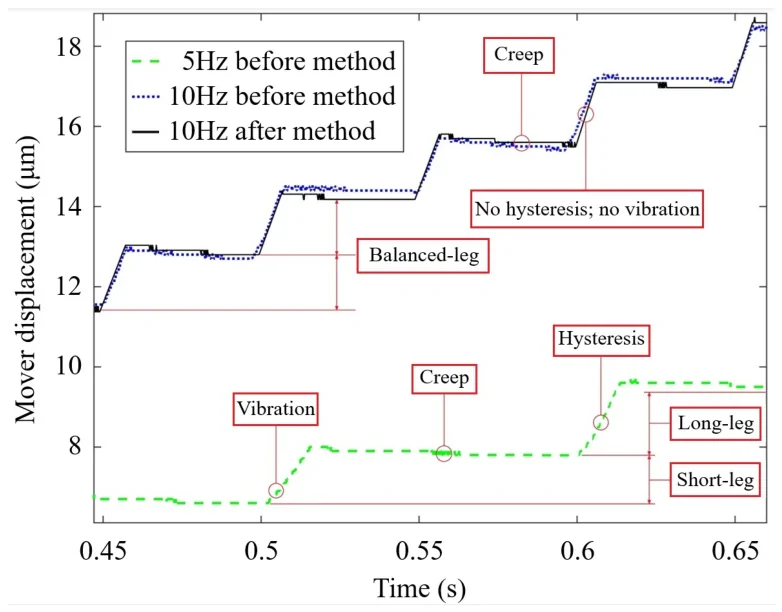
Enlarged comparison of displacement.

Figure 8 presents an enlarged view of Figure 7. The adjusted 5 Hz displacement is omitted to facilitate identification of the “long-leg/short-leg” phenomenon in the unadjusted displacement. Comparing the green long-dashed 5 Hz displacement with the blue dash-dotted 10 Hz displacement before adjustment shows that the hysteresis and “long-leg/short-leg” characteristics of the forward mover motion persist regardless of frequency. In addition, vibration occurs during forward motion, which may be attributed to relative sliding caused by the previously discussed nonuniform velocity.

The 10 Hz displacement response after adjustment, represented by the black solid line, shows a substantial reduction in the apparent hysteresis and the “long-leg/short-leg” displacement discrepancy during forward motion. This indicates that the differences in material actuation capability and nonlinear characteristics of the shear stacks have been effectively compensated. Both groups of displacement curves in the enlarged plot exhibit a retraction displacement after forward motion. This is attributed to the increase in preload caused by creep of the longitudinal stacks, which reduces the actual output of the shear stacks while maintaining the mover position [27]. Since creep was not included within the compensation range in three-stage voltage adjustment method, the observed retraction displacement can be reasonably explained. Table 2 compares the mean displacement, retraction displacement, and net displacement of the “long-leg” and “short-leg” conditions before and after adjustment at 10 Hz. In this experiment, the net displacement difference rate decreased from 32.32% to 3.60%, corresponding to an absolute reduction of 28.72 percentage points. This result indicates improved single-step displacement consistency for the tested inchworm piezoelectric structure under the specified operating conditions.

The above improvement is based on the measured data from the reported experiment. Since independent repeated trials and statistical uncertainty estimates were not obtained, the result should not be interpreted as a statistically validated improvement applicable to all devices or operating conditions. Nevertheless, the measured displacement curves and the corresponding single-step displacement comparison provide experimental evidence that the proposed voltage-adjustment procedure can reduce the observed actuation inconsistency in the tested motor.

It is worth noting that the voltage scaling adjustment and contact timing adjustment of the longitudinal stacks also improve the symmetry of the contact motion. The consistency of the preload causes the slopes of the creep-induced retraction to become more similar. Therefore, the retraction slopes after adjustment appear relatively balanced in the figure, whereas the retraction slopes before adjustment exhibit substantial differences.

Overall, the displacement experiments designed for the inchworm piezoelectric structures demonstrate that the three-stage voltage adjustment method effectively improves the stability of mover’s motion, particularly the action consistency of the inchworm piezoelectric structures. The adjustment improves the velocity uniformity of the macro-stage driven by periodic motion, effectively reduces the stopping-position error associated with individual steps, and improves motion continuity.

## 5. Conclusions

This study addresses the inconsistent action problem of inchworm piezoelectric structures used in the macro stage of tea-coffee nanoscale detection device and proposes a three-stage voltage adjustment method. The method consists of three progressive stages: actuation capability correction, shear nonlinear compensation, and opposite-side consistency alignment, which respectively address the three identified aspects of piezoelectric elongation, velocity consistency, and contact timing. Experimental results show that after the three-stage adjustment, the net displacement difference rate of the mover drived by inchworm piezoelectric structure is reduced from 32.32% to 3.60%, demonstrating the effectiveness of the proposed method in correcting action inconsistency.

The proposed three-stage voltage adjustment method improves the observed single-step displacement consistency and motion continuity of the tested inchworm piezoelectric structure without requiring modifications to its mechanical configuration or driving connections. The method is intended as a practical engineering procedure for correcting actuation-capability differences, compensating for shear hysteresis, and aligning contact timing. However, the present method is a stage-wise compensation framework rather than a complete model of all nonlinear effects in inchworm piezoelectric motion. In particular, creep-induced displacement retraction remains outside the current compensation procedure. Other operating strategies may also require additional voltage regulation mechanisms to account for creep and other time-dependent effects. Furthermore, the adjustment procedure relies on offline calibration and does not currently provide online adaptive parameter updating. The reported displacement improvement is based on the available experimental measurements, hence further repeated experiments are needed to establish statistical repeatability including long-duration cycling. These extensions will be considered when the compensation architecture is further developed.

## Figures and Tables

**Table 1:** Experimental driving-voltage parameters.

Type	Voltage Amplitude (V)	Frequency (Hz)	Actuate (Clamp)	Reset (Release)
Longitudinal stack	120	5, 10	0.125	0.875
Shear stack	240	5, 10	0.125	0.875

**Table 2:** Comparison of 10 Hz single-step displacement before and after adjustment (unit: μm).

Measured displacement	Before Adjustment	After Adjustment
“Long-leg” displacement	1.86	1.61
“Long-leg” retraction	0.22	0.22
“Long-leg” net displacement	1.64	1.39
“Short-leg” displacement	1.43	1.60
“Short-leg” retraction	0.32	0.26
“Short-leg” net displacement	1.11	1.34
Final displacement at 10 s	275.3 (expected)	272.7
Net displacement difference rate	32.32%	3.60%

## Data Availability

Restrictions apply to the availability of these data. The data were obtained from the China Postdoctoral Science Foundation (2025T180138) and are available with permission.
